# Complement System Dysregulation in the Immunopathogenesis of Long COVID: Systematic Evidence Synthesis

**DOI:** 10.3390/biomedicines14020439

**Published:** 2026-02-15

**Authors:** Kin Israel Notarte, Jesus Alfonso Catahay, Jacqueline Veronica Velasco, Abbygail Therese Ver, Jungwook Lee, John G. Rizk, Giuseppe Lippi, César Fernández-de-las-Peñas

**Affiliations:** 1 Department of Pathology, Johns Hopkins University School of Medicine, Baltimore, MD 21205, USA; knotart1@jhu.edu; 2 Division of Nephrology, Montefiore Medical Center, Albert Einstein College of Medicine, New York, NY 10461, USA; jesus.catahay@gmail.com; 3 Department of Internal Medicine, Cardinal Santos Medical Center, San Juan City, Metro Manila 1502, Philippines; jacqvelasco96@gmail.com; 4 Alliance for Improving Health Outcomes, Quezon City, Metro Manila 1104, Philippines; abbygailver@gmail.com; 5 Department of Biology, Johns Hopkins University, Baltimore, MD 21218, USA; jlee785@jhu.edu; 6 Department of Practice, Sciences, and Health Outcomes Research, School of Pharmacy, University of Maryland, Baltimore, MD 21201, USA; john.rizk@umaryland.edu; 7 Section of Clinical Biochemistry, University of Verona, 37129 Verona, Italy; giuseppe.lippi@univr.it; 8 Department of Physical Therapy, Occupational Therapy, Physical Medicine and Rehabilitation, Universidad Rey Juan Carlos, 28922 Madrid, Spain

**Keywords:** complement, immune dysregulation, long COVID, post-COVID-19 condition

## Abstract

**Background/Objective:** Long COVID is an important cause of disability following SARS-CoV-2 infection; yet, its underlying mechanisms are not completely understood. One proposed mechanism is the long-lasting dysregulation of the immune complement system. This systematic review is the first to summarize the current evidence and evaluate the potential role of long-lasting complement activation in people with long COVID. **Methods:** A systematic electronic search on PubMed, MEDLINE, CINAHL, and Embase was conducted up to 15 October 2025, to identify studies investigating complement activation in people with the post-COVID-19 condition. The Newcastle–Ottawa Scale was used to evaluate the risk of bias and methodological quality. **Results:** Among the 247 studies initially identified, eleven met the inclusion criteria, comprising 1435 individuals (age: 48.5 years, 70% females) with long COVID and 1124 controls (age: 43.6 years, 60% females). All studies were of a high quality, with scores ranging from 7 to 8 stars (mean: 7.6 ± 0.5). The activation of the classical complement pathway was investigated in nine studies, whereas the lectin, alternative, and terminal complement pathways were each assessed in three studies. Multiple studies investigated several complement pathways. The results were heterogeneous since several markers of complement activation spanning the classical (C2, C4a, C4b, and C1s-C1INH), alternative (Ba, iC3b, and Factor D), and terminal (C5bC6, C5a, C9, and TCC) pathways were elevated, whereas other markers were not significantly different (C3, C4, and C4d) between patients with/without long COVID. In addition, markers spanning the lectin complement pathway (MBL, and MASP1-C1INH) were not significantly different between individuals with and without long COVID. **Conclusions:** The current evidence suggests potential long-lasting complement system dysregulation in individuals with long COVID, although the clinical significance remains controversial, due to heterogenous findings. Specific post-COVID symptom clusters, such as fatigue, dyspnea, or brain fog, have been linked to a distinct pattern of complement dysregulation. Substantial methodological heterogeneity, including differences in follow-up periods, complement markers, assessment methods, and control groups, along with the small number of available studies, underscores the need for further research to clarify the mechanisms linking complement dysregulation to long COVID.

## 1. Introduction

**Figure 1:**
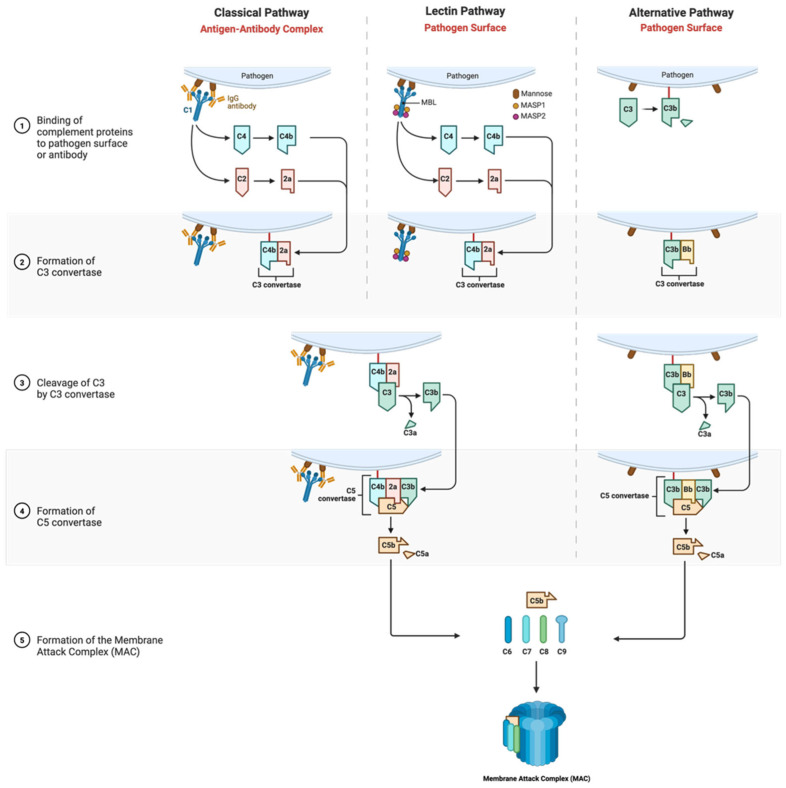
Classical, lectin, and alternative pathways of the complement system. MBL: mannose-binding lectin; MASPs: MBL-associated serine proteases.

Long COVID, also known as post-acute COVID-19 syndrome, is characterized by persistent symptoms after the acute phase of a SARS-CoV-2 infection [1]. Commonly reported features include fatigue, dyspnea, cognitive dysfunction, and chest pain [2]. Epidemiological studies estimate that a substantial proportion of individuals experience symptoms after the initial infection, with recent data demonstrating persistence for up to two to three years [2,3]. The underlying mechanisms remain incompletely defined, but several hypotheses, including sustained organ damage, the persistence of viral RNA or proteins, the generation of autoantibodies, the reactivation of latent viruses, and chronic immune or endothelial dysfunction, have been proposed [4,5,6,7]. The complement system is an essential feature of the innate immunity that contributes to pathogen recognition, cell lysis, and the regulation of inflammation [8]. Its activation occurs throughout the classical, lectin, and alternative pathways, which are activated differently but converge to amplify inflammatory responses and generate the membrane attack complex (MAC) [9]. Specifically, the classical pathway is activated by antigen–antibody complexes, the lectin pathway by pathogen-associated carbohydrate patterns, and the alternative pathway by spontaneous activation on microbial or altered host cell surfaces (Figure 1). Regardless of the initiating stimulus, all three pathways converge at C3, leading to C5 cleavage and activation of the terminal pathway, culminating in MAC formation. In the lectin pathway, mannose-binding lectin (MBL) binds to MBL-associated serine proteases (MASPs) to initiate downstream complement activation. Collectively, these tightly regulated processes enable effective immune defense against infection while preserving host tissue integrity.

The complement system is also closely interconnected with the circulatory coagulation system, and its dysregulated activity can promote thrombo-inflammation and tissue injury [10]. Excessive complement activation has been associated with endothelial damage, microvascular thrombosis, or severe outcomes during the acute COVID-19 phase [11]. Emerging evidence suggests that complement dysregulation may also persist in individuals with long COVID, where ongoing inflammation, coagulation abnormalities, and vascular injury are present [12].

To date, no systematic review has synthesized the available evidence on the role of the complement cascade in the immunopathogenesis of long COVID. The objective of this systematic review was to address this gap by evaluating the current data and clarifying the potential contribution of long-lasting complement activation to the persistence of post-COVID-19 symptoms.

## 2. Methods

A systematic review of studies investigating differences in complement activation in people with post-COVID symptoms according to the 2020 Preferred Reporting Items for Systematic Reviews and Meta-Analyses (PRISMA) statement was conducted (Supplementary Materials) [13]. The review was registered in the Open Science Framework database (https://osf.io/6s45h). A meta-analysis was not conducted because studies are highly heterogenous in the complement markers assessed, the assays and biological measures used (intact complement components, activation fragments, functional assays, and omics-derived pathway signals), baseline values, follow-up timeframes, and post-COVID outcomes evaluated.

### 2.1. Literature Search

Literature searches for studies published up to 15 October 2025 were conducted across four databases: PubMed, Medline, CINAHL, and Embase. An experienced health science librarian assisted with the electronic searches. The reference lists of identified studies were also screened for additional studies to be included. Search terms and Boolean operators used in the literature search on each database are outlined in Table 1.

### 2.2. Selection Criteria

The inclusion criteria were described according to the Population, Intervention, Comparison, and Outcome (PICO) principle:

Population: Adults (>18 years) who had been previously infected by SARS-CoV-2;
Intervention: Not applicable;
Comparison: Not applicable;
Outcome: Articles investigating complement activation in individuals with post-COVID-19 condition. We used the definition by Soriano et al.: “post-COVID-19 condition occurs in people with a history of probable or confirmed SARS-CoV-2 infection, usually three months from the onset of infection, with symptoms that last for at least two months and cannot be explained by an alternative diagnosis” [14]. Articles should report post-COVID symptoms as well as measure complement activation at any follow-up period post-infection.

### 2.3. Screening Process, Study Selection, and Data Extraction

Cohort and case–control studies where complement activation in patients who developed post-COVID-19 condition after an acute SARS-CoV-2 infection were included. Papers that are not original articles, such as systematic reviews or meta-analyses, and editorials, were excluded. Studies were also excluded if the samples were not collected from humans. Post-mortem studies were excluded.

The titles and abstracts of each article identified during the literature search were screened by two authors. After removing duplicates, the full text of eligible articles was analyzed by the same authors. For each study, the following data were extracted: authors and country, sample size and setting, participant characteristics (age, sex, demographic, anthropometric, and clinical data), post-COVID symptoms, sample collection methods, diagnostic methods used to assess complement activation, and follow-up duration. Consensus on the selection of included studies and on data extraction should be achieved by both authors. Discrepancies at any stage of the screening process were resolved by asking a third author, if needed.

### 2.4. Methodological Quality/Risk of Bias

The Newcastle–Ottawa Scale (NOS) was used to evaluate the risk of bias and methodological quality of the included observational studies [15]. Scores were classified as follows: high-quality (7–9 stars), moderate-quality (5–6 stars), and low-quality (≤4 stars). Methodological quality was independently evaluated by two authors, and any discrepancies were resolved through discussion with a third researcher.

## 3. Results

### 3.1. Study Selection

**Figure 2:**
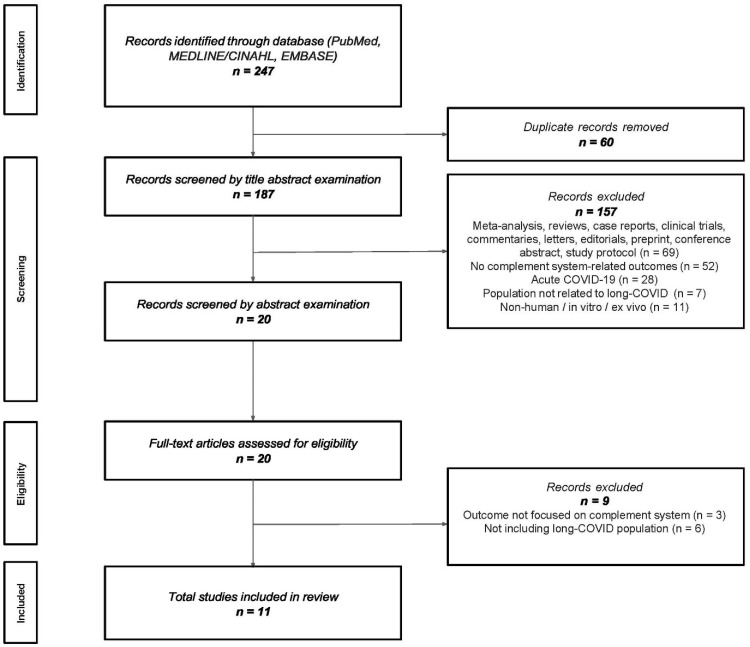
The PRISMA flow diagram.

The database search initially identified 247 studies. After removing 60 duplicates, 187 studies remained for the title and abstract examination, and then 157 studies were excluded at the title screening because of the type of paper (e.g., meta-analyses, reviews, case reports, clinical trials, commentaries, and editorials), population (acute COVID-19 patients or other population different from long COVID), measurements (not related to the complement system), or the papers being non-human studies. Then, 20 studies underwent an abstract and detailed full-text review. Among these, 9 were further excluded: 3 studies did not assess the complement system biology, and the remaining 6 did not include a definition of the long COVID population. Overall, eleven studies met all the eligibility criteria and were included in the review [12,16,17,18,19,20,21,22,23,24,25]. Figure 2 describes the PRISMA flow diagram.

### 3.2. Sample Characteristics

A total of 1435 individuals (mean age: 48.5 years old, 70% females) with long COVID and 1124 controls (mean age: 43.6 years, 60% females) were included in the studies. We found 5 studies included hospitalized COVID-19 survivors [12,19,20,21,25], 2 included non-hospitalized survivors [18,22], and the remaining 4 [16,17,23,24] did not specify the participants’ hospitalization status. The most common self-reported post-COVID symptoms included fatigue, dyspnea, brain fog, headache, insomnia, dysgeusia, hair loss, poor concentration, dizziness, sleep disturbance, or gastrointestinal problems (diarrhea, vomiting, and abdominal pain). All studies measured the complement proteins in serum or plasma, but the methods differed, including ELISA [12,18,21,22,23], mass spectrometry [12,17], immunoassay/proteomics [12,19], and other assay platforms [16,17,20,24,25]. Samples were also collected at different time points after the infection, ranging from three months to two years after SARS-CoV-2 infection (mean follow-up: 8.9 months). Table 2 summarizes the main findings of the studies included in this review.

### 3.3. Methodological Quality

Seven case–control (Table 3) [17,18,19,20,22,25] and four longitudinal cohort (Table 4) [12,16,21,23,24] studies were evaluated. All studies were of a high methodological quality, with scores ranging from 7 to 8 stars (7.6 ± 0.5). No disagreements were observed between the two authors.

### 3.4. Classical Complement Pathway in Long COVID

The activation of the classical complement pathway was investigated in nine studies [12,16,17,18,19,20,21,22,25] with heterogeneous results. For instance, elevated C2 and C5 protein levels were reported at six [12] and twelve [17] months after infection, whereas no significant differences in C3, C4, and C5 levels were observed at one [18] and two [22] years post-infection among survivors with long COVID. Similarly, elevated levels of C4a and C4b activation products were observed in patients with long COVID compared to pre-infected controls without long COVID at one-year post-infection [17,19]. In contrast, no differences in C4d product levels between people with long COVID, non-previously infected individuals, and pre-infected controls without long COVID were identified six months after infection [12]. Wei et al. observed that subjects with long COVID showed significant dysregulation in the complement cascade including C1qA, C1qC, or C4a activation products as well as C3 and C5 components compared to patients without long COVID, but these authors did not report whether this dysregulation reflected increased or decreased complement levels [25]. Baillie et al. [18] observed increased levels of the C1s/C1-inhibitor (C1s-C1INH) complex in individuals with long COVID compared with matched (by age/ethnicity/sex/infection/vaccination status) infected controls without long COVID one year after infection; however, Hurler et al. found no differences in the C1-INH product, the C1s/C1-INH complex, or pentraxin 3 (PTX3) levels among patients with long COVID, previously infected controls without long COVID, and non-infected controls; infected participants were evaluated at ~90 days post-infection [21].

Contradictory results in the same component of the classical pathway have also been reported. Aschamn et al. observed elevated levels of C1q in long COVID [17], Baillie et al. reported lower levels of C1q [18], and Hurler et al. did not find significant differences in C1q [21] between individuals with and without long COVID. It should be noted that Aschamn et al. [17] and Baillie et al. [18] measured the C1q component levels one year after infection, whereas Hurler et al. [21] evaluated C1q at approximately three months post-infection.

Further, heterogeneity in the data reported according to specific post-COVID symptoms was noted. For instance, Cervia-Hasler et al. identified increased levels of the C2 component in patients experiencing post-COVID fatigue one year after infection [12], whereas Fernández-de-las-Peñas et al. observed decreased levels in the C3 and C5 components among survivors with post-COVID fatigue or dyspnea two years post-infection [22]. Liew et al. also observed increased levels of the C1qA activation product in individuals with gastrointestinal or cognitive post-COVID symptoms, but not in those with fatigue or cardiorespiratory symptoms [20]. Similarly, Hagiya et al. analyzed the CH50 level as a global measure of the functional activity of the classical complement pathway and reported a significant association between higher CH50 levels and post-COVID brain fog (adjusted OR 1.66, 95%CI 1.04–2.66), but not post-COVID fatigue or dyspnea, at three months post-infection [16].

### 3.5. Lectin Complement Pathway in Long COVID

The activation of the lectin complement pathway was investigated in three studies [12,18,23], yielding heterogeneous findings. Cervia-Hasler et al. observed no differences in MBL levels, a central component of the lectin pathway, between individuals with long COVID and infected and non-infected controls [12]. Similarly, no significant difference in the serine protease 1 (MASP1)-C1INH complex was observed by Baillie et al. [18]. On the contrary, Bulla et al. reported lower MBL levels in patients experiencing post-COVID brain fog than in long COVID survivors without brain fog and with healthy (non-infected) controls [23].

### 3.6. Alternative Complement Pathway in Long COVID

The activation of the alternative complement pathway was investigated in three studies [18,20,24]. Baillie et al. observed higher levels of Ba and iC3b activation products as well as increased levels of regulators Factor D and properdin, while the Factor B levels were similar in individuals with long COVID compared with infected controls without long COVID [18]. On the contrary, Song et al. showed a decrease in a specific complement component (C3f) following a first or second SARS-CoV-2 infection [24]. It should be noted that C3f is a fragment resulting from the cleavage of the C3 protein, which is crucial for all (classical, lectin, and alternative) complement pathways. Liew et al. observed increased levels of COLEC12 in people with post-COVID fatigue and cardiorespiratory symptoms [20].

### 3.7. Terminal Complement Pathway in Long COVID

Finally, the terminal pathway was investigated in three studies [12,18,22]. Cervia-Hasler et al. found increased levels of the C5bC6 complex and decreased levels of the C7 protein in individuals with long COVID at six months post-infection [12]. Age and vaccination status were not associated with C7 protein levels [12]. Baillie et al. observed increased levels of the C5a activation product, as well as higher levels of the C9 component and terminal complement complex (TCC) in people with long COVID as compared to infected controls without long COVID [18]. On the contrary, Fernández-de-las-Peñas et al. found no overall differences in C7 protein levels between survivors with and without long COVID at two years post-infection [22]. Patients experiencing post-COVID dyspnea showed a decrease in C7 protein levels as compared to those without post-COVID dyspnea [22].

## 4. Discussion

The complement system is a crucial component of the innate immune system since it serves as the first line of defense against infection and facilitates immune responses. When activated, the complement system triggers a cascade of inflammatory reactions vital for immune surveillance and homeostasis. The protective capacity of this system relies on a delicate balance between its various factors and regulators. A disturbance in this network causes significant damage to the host tissue, as it is amplified by the nonspecific, target-driven cascade and further fueled by interactions with other immune and homeostatic pathways. Multiple studies have shown complement dysregulation among COVID-19 patients as a major driver of disease pathology at the acute phase of infection [11]. While it is still unclear how complement dysregulation can be sustained for long periods of time, this was originally attributed to the viral persistence in tissue reservoirs after the acute infection [26]. Nevertheless, the presence of long-lasting viral persistence in people with long COVID has not been consistently confirmed [27,28]. Additionally, since persistent inflammation has been proposed as a mechanism of long COVID and given the role of the complement system in inflammation, the persistent chronic inflammatory state found in people with the post-COVID-19 condition could lead to the long-lasting activation of the complement system [29]. In such a scenario, elevated levels of the complement system components are indicative of acute inflammation and tissue damage, whereas decreased levels of complement system components reflect uncompensated consumption potentially associated with the prolonged and unbalanced hyperactivation of the immune system [30]. Thus, the complement system has a dual role in host autoimmune propensity since its deficiency prevents the proper clearance of debris and triggers autoimmunity whereas its overactivation causes chronic inflammation. This systematic review included eleven studies investigating the complement system in individuals with long COVID. The results suggest a dysregulated complement system in individuals with long COVID; however, the findings were heterogenous, not only in the results (some studies showed increased levels, others decreased levels, and others no differences; Table 5), but also in the follow-up periods (three, six, twelve, and twenty months), as well as in complement pathways (e.g., classic, lectin, alternative, or terminal) investigated and the diagnostic methods used.

### 4.1. Complement System Dysregulation in Long COVID

Based on the available evidence, the current review of the literature suggests the following: (1) several markers of complement activation spanning the classical (C2, C4a, C4b, and C1s-C1INH), alternative (Ba, iC3b, and Factor D), and terminal (C5bC6, C5a, C9, and TCC) pathways were generally elevated in patients with long COVID compared with infected subjects without long COVID, whereas other markers (C3, C4, and C4d) were not significantly different between groups; and (2) immune markers spanning the lectin complement pathway (MBL, and MASP1-C1INH) were generally not significantly different between subjects with and without long COVID.

The results on the classical pathway were highly heterogeneous since increased levels of C2 and C5 components, C4a and C4b activation products, and C1s/C1-inhibitor (C1s-C1INH) complex were found in individuals with long COVID [12,17,18,19], whereas no differences in the C3, C4, and C5 components, C4d product, C1s/C1-INH complex, or PTX3 [12,18,21,22] were identified. Contradictory results on the same (C1q) component were also found. Aschamn et al. observed elevated levels of the C1q component [17], whereas Baillie et al. reported lower levels of C1q [18] in individuals with long COVID one year after the infection. In addition, one study suggested dysregulation of the complement cascade involving C3 and C5, as well as C1qA, C1qC, and the activation product C4a, in individuals with long COVID; however, it did not report the direction of change (increased vs. decreased) for these immune markers [25]. Interestingly, reduced levels of the C1q component have been associated with autoimmune diseases such as systemic lupus erythematosus [31]. Accumulating evidence suggests that SARS-CoV-2 infection can lead to a broad spectrum of immune-mediated complications, ranging from well-defined autoimmune conditions to the development of diverse autoantibodies and atypical inflammatory phenotypes [32]. However, the heterogeneous findings observed in the current review do not allow a clear determination of whether changes in the classical complement pathway primarily reflect an increased autoimmune propensity associated with lower complement levels or, instead, reflect acute immune responses during infection associated with higher complement levels.

In relation to the alternative complement pathway, individuals with long COVID showed higher levels of Ba and iC3b activation products, and higher levels of Factor D, but similar levels of Factor B [18] when compared with infected controls without long COVID. Nevertheless, Song et al. observed that the C3f component levels decreased with consecutive infections, suggesting the progressive, uncompensated consumption of this product associated with the cleavage of the C3 component [24]. Data on the terminal component pathway were heterogeneous since increased levels of the C5bC6 complex, C5a activation product, C9 component, and TCC, but lower levels of the C7 protein were observed in people with long COVID as compared to infected controls without long COVID [12,18]. Another study investigating markers of the terminal pathway revealed no differences in C7 protein levels between individuals with and without long COVID [22].

Finally, findings for the lectin pathway were relatively consistent, with two studies reporting no between-group differences in MBL or MASP1-C1INH levels among survivors with/without long COVID [12,18]. Overall, the data suggests that different complement pathways may respond differently to SARS-CoV-2 infection.

An important observation of the current review is the heterogeneous follow-up periods of the included studies leading to a potential “time–complement pathway interaction”. The fluctuating nature of the complement system is supported by two studies. Hurler et al. observed that C1q product levels were increased during the active phase of infection (days 0 and 14), but not at days 28 or 90 post-infection [21]. Similarly, Song et al. observed a gradual decrease of the complement component C3f during the recovery period after the first and second infections [24]. Hence, it is possible that the complement system is activated during the acute phase of COVID-19 as part of the natural immune response against SARS-CoV-2, leading to increased levels of complement components [11]. In individuals with persisting symptoms, sustained or excessive complement activation may result in higher levels of complement proteins and activation products, potentially reflecting ongoing immune activation and a persistent inflammatory state involving macrophages and dendritic cells [29]. In a later, prolonged stage, sustained complement hyperactivation may ultimately result in reduced circulating complement proteins and activation products due to the ongoing, uncompensated consumption over time. Longitudinal analyses within the same cohort are needed to confirm whether complement responses fluctuate across phases of long COVID.

### 4.2. Complement System and Post-COVID Symptomatology

Some studies have suggested that complement system dysregulation could be symptom-specific; however, the current evidence is heterogeneous due to the assessment of multiple components or activation products from different complement pathways. For example, among survivors reporting post-COVID fatigue, C2 levels were higher at six months post-infection [12], C3 levels were lower at two years [22], and CH50 levels at three months [16], and C1qA activation products at six months [20] were not different from controls. In addition, survivors experiencing post-COVID fatigue exhibited higher levels of COLEC12 (a molecule that activates the alternative complement pathway, specifically by binding to properdin independent of C3b) at six months post-infection than those without fatigue [20]. Similarly, individuals with post-COVID dyspnea showed lower levels of C3, C5, and C7 at two years post-infection [22], but had normal CH50 levels at three months post-infection [16]. The most consistent findings were observed among individuals with post-COVID brain fog: higher CH50 levels were reported at three months [16], and increased C1qA activation products at six months [20]. The differences across studies may reflect the limited number of available studies and small sample sizes. Phenotyping post-COVID patients by symptom clusters [33] may help define more homogeneous study groups for evaluating complement dysregulation in future works.

### 4.3. Limitations

Although most studies support a dysregulation of the complement system in people with long COVID, the findings should be interpreted cautiously. First, the evidence is based on heterogeneous study designs, small sample sizes (n < 50) in several studies [17,22,23,24,25], and different follow-ups, which restrict the temporal inference about complement system dysregulation. Second, control groups were not uniform across studies; some comparison groups included participants who had recovered from a SARS-CoV-2 infection without developing long COVID or controls who had no prior acute infection, creating a potential selection bias. Third, no study established pre-infection baseline levels or even levels of the complement system at the acute COVID-19 phase, making it difficult to identify post-infection changes from the pre-existing inter-individual variation. Fourth, the post-COVID symptomatology assessment relied on self-report with limited harmonization, raising the possibility of misclassification and weakening links between immunologic findings and clinical phenotypes. Fifth, most studies included adult populations from different clinical settings (i.e., hospitalized, non-hospitalized, or non-specified), limiting generalizability and precluding the inference to adolescents/children. Sixth, although all studies included were considered to be of a high methodological quality, it should be considered that measurement bias is not an item included in the Newcastle–Ottawa Scale. In fact, the use of different pre-analytic handling (e.g., serum vs. plasma, processing delay, freeze–thaw, and ex vivo activation) and of multiple assessment methods (e.g., ELISA assays, mass spectrometry, and proteomics), with a different sensitivity and specificity, may explain the heterogeneity and comparability of the results.

Finally, a key limitation of the published studies is that, despite the known effects of sex, age, and obesity (BMI) on complement biology, the adjustment for these factors was inconsistent. In fact, only Baillie et al. considered sex, age, and BMI as biological confounders in their analyses [18]. Females were reported to exhibit differences in complement levels and functionality compared with males, particularly in the alternative complement pathway. Gaya da Costa et al. observed that females exhibit lower levels of the C3 component and properdin regulator, and a higher factor D concentration than males, but without differences in the classical pathway activity [34]. Only Baillie et al. included sex as a confounder factor and found no effect on the differences observed in the alternative pathway between individuals with and without long COVID [18]. It should be noted that most studies did not report sex-stratified data or consider sex as a potential biological confounder. As a result, women comprised up to 70% of long COVID participants, and meaningful sex-based comparisons were not possible with the available data. In addition, aging has been associated with increased complement pathway activity, particularly in the classical and alternative pathways, but not the lectin pathway, which may contribute to higher levels of activation products (e.g., C3a and C5a) and complement components (e.g., C3, C4, and C5) [35]. Most samples of patients included in the current review were middle-aged adults (between 40–50 years), so the effect of age would be minimal. In fact, Cervia-Hasler et al. [12] and Baillie et al. [18] did not find a significant effect of age on the complement system levels. Obesity induces a state of chronic, low-grade inflammation that activates the complement system, particularly the classical pathway, by triggering the production of C3 and C1q components [36]. Only Baillie et al. adjusted for BMI as a confounder and found that it did not affect the observed differences in the alternative pathway between individuals with and without long COVID [18]. Because sex, age, and BMI were not consistently accounted for in most studies, despite their known influence on complement biology, the effect estimates may be subject to bias.

Accordingly, these limitations preclude causal conclusions about the role of complement system dysregulation in people with long COVID and highlight the need for adequately powered longitudinal studies with the standardized phenotyping of the samples, clearly defined comparator groups, baseline measurements where feasible, and, at least, a proper matching and adjustment for sex, age, and BMI.

## 5. Conclusions

This systematic review discusses the current insights into the relationship between complement activation and long COVID. While the evidence suggests a potential association between long COVID and complement system dysregulation, the clinical significance remains controversial due to the heterogeneous findings. Overall, abnormalities have been more consistently reported in the classical, alternative, and terminal pathways than in the lectin pathway, although the responses appear to vary across studies and timepoints. In addition, specific post-COVID symptom clusters, such as fatigue, dyspnea, or brain fog, have been linked to a distinct pattern of complement dysregulation. The limited number of studies, methodological variability, differences in study populations and control groups, heterogeneity in long COVID symptom profiles, and the lack of pre-infection baseline complement measurements underscore the urgent need to clarify whether complement dysregulation is truly associated with long COVID and to identify the mechanisms that may underline any such association. Further studies acknowledging these gaps are now essential for tailored diagnostic and treatment approaches to enhance patient care and outcomes in the context of long COVID. 

## Figures and Tables

**Table 1:** Database formulae during literature search.

PubMed Search Formula
1 “post-acute COVID-19 syndrome” [All Fields] OR “long COVID” [All Fields] OR “long COVID symptoms” [All Fields] OR “long hauler” [All Fields] OR “post-COVID 19” [All Fields] OR “post-acute COVID-19 symptoms” [All Fields] OR “COVID-19 sequelae” [All Fields] 2 “complement system” [All Fields] OR “complement markers” [All Fields] OR “complement activation” [All Fields] 3 #1 AND #2
Medline/CINAHL (via EBSCO) Search Formula
1 (post-acute COVID-19 syndrome) OR (long COVID) OR (long COVID symptoms) OR (long hauler) OR (post-COVID 19) OR (post-acute COVID-19 symptoms) OR (COVID-19 sequelae) 2 (complement system) OR (complement markers) OR (complement activation) 3 #1 AND #2
Embase Search Formula
[(post-acute COVID-19 syndrome) OR (long COVID) OR (long COVID symptoms) OR (long hauler) OR (post-COVID 19) OR (post-acute COVID-19 symptoms) OR (COVID-19 sequelae)] AND [(complement system) OR (complement markers) OR (complement activation)]

**Table 2:** Main findings on complement activation in long COVID among included studies.

Author Country	Study Design Sample Size	Study Timeline	Mean (Range/±SD) Age Females (%)	Clinical Data	Post-COVID Symptoms	Sample Analyzed Assessment Method	Follow-Up Period	Main Findings
Cervia-Hasler et al. (2024) [12] Switzerland & USA	Prospective cohort Zurich Cohort Healthy Controls (n = 39) Without LC (n = 73) With LC (n = 40) Mount Sinai Cohort Healthy controls (n = 35) Controls hospitalized not for COVID-19 (n = 47) COVID-19 survivors (n = 198)	Zurich cohort April 2020-April 2021 Mount Sinai April–June 2020	Zurich Cohort Controls 38 (28–54) y 56.4% females Without LC 41 (28–59) y 45.2% females With LC 56 (42–69) y 52.5% females Mount Sinai Cohort Controls 55 (20–90) y 50% females With LC 56 (42–69) y 43% females	Hospitalized COVID-19 Severity Severe: n = 33 BMI: N/A	Cough, dyspnea, fatigue, gastrointestinal problems, headache, chest pain, smell/taste disorder, tachycardic, neuropathic symptoms, joint/muscle pain, and muscle weakness	Serum ELISA, aptamer-based proteomics, functional complement assay, and mass spectrometry	One year after the infection	↑ C2, ↑ C5bC6 and ↓ C7 levels in patients with LC. No difference in C4d and MBL levels between patients with/without LC and healthy controls.
Aschman et al. (2023) [17] Germany	Case–control Without LC (n = 15) With LC (n = 9)	June 2020–November 2021	People without LC 42.6 ± 10.9 y 90% females People with LC 45.1 ± 11.5 y 90% females	COVID-19 Severity: Mild: n = 10 Severe: n = 1 BMI: N/A	Loss of taste/smell, headache, fatigue, dyspnea, myalgia, arthralgia, rhinitis, chest pain/tightness, sore throat, cough, cognitive impairment, vertigo, and post-exertional malaise	Serum Bulk RNA-seq, immunohistochemistry, and mass spectrometry	369 ± 97 days after the infection	↑ C1qA, C1qB, C1q C, C4a, and C5 levels in patients with LC as compared to those without LC.
Baillie et al. (2024) [18] United Kingdom	Case–control Without LC (n = 79) With LC (n = 166)	March 2020–October 2022	People without LC 45 (21–82) y 78.5% females People with LC 47 (20–83) y 76.5% females	Non-hospitalized COVID-19 Severity: N/A BMI >30 kg/m^2^ (n) Without LC (n = 26) With LC (n = 79)	Breathlessness, fatigue, musculoskeletal problems, neuropsychiatric problems, and pain	Plasma ELISA and functional hemolytic assay	At least 3 months (90 days) after infection	↑ C1s-C1-INH complex, Ba, iC3b, C5a, C3, C5, C9, Factor D, TCC Properdin, levels and ↓ C1q in LC patients. No differences in C4 and Factor B levels.
Bulla et al. (2023) [23] Italy	Case–control Healthy Controls (n = 18) With LC (n = 48) No brain fog (n = 16) Brain fog (n = 32)	November 2021–March 2022	Healthy Controls 43.5 (28.5–53.5) y 72.2% females No Brain fog 46.5 (31.7–62.2) y 75% females Brain fog: 53.5 (44.75–58.25) y 75% females	COVID-19 severity: N/A BMI: Obesity (n) Controls (n = 4) With LC (n = 8)	Fatigue, dyspnea, myalgia/arthralgia, hyposmia/ hypogeusia, insomnia, headache, mood disorders, paresthesia, ocular problems, tinnitus, dizziness, gastrointestinal symptoms, tachycardia, and palpitations	Serum ELISA and Wieslab^®^ functional pathway assay	320 (279–367) days after infection	Patients with post-COVID brain fog had ↓ levels of MBL than those without brain fog. Low MBL levels (%): With brain fog (31.3%); Without brain fog (18.8%); Healthy controls (22.2%).
Fernández-de-las-Peñas et al. (2025) [22] Spain	Case–control Without LC (n = 27) With LC (n = 57)	Not Reported	Mean age ± SD Without LC 46.5 ± 11.5 y 55% females With LC 46.5 ± 9.0 y 56% females	Non-hospitalized COVID-19 severity: N/A BMI: N/A	Fatigue, dyspnea, brain fog, pain, memory loss, ageusia, anosmia, gastrointestinal problems, concentration loss, ocular problems, hair loss, palpitations, diarrhea, fever, myalgia, cough, headache, throat pain, and skin rashes	Serum ELISA, CH50 hemolytic assay, and immunochemical assay	Without LC 2 ± 1.7 years after infection With LC 1.7 ± 1.2 years after infection	No differences in C3, C4, C5, C7, or CH50 levels between subjects with and without LC. Patients with fatigue had ↓ C3 than those without fatigue. Patients with post-COVID dyspnea had ↓ C3, C5, and C7 than those without dyspnea.
Hagiya et al. (2024) [16] Japan	Retrospective cohort Normal CH50 (n = 194) High CH50 (n = 284)	February 2021–March 2023	Normal CH50: 41 (26–51) y 52.6% females High CH50 (≥59 U/mL): 44.5 (35.75–52.25) y 54.2% females	COVID-19 severity: Mild (n = 404) Moderate (n = 29) Severe (n = 45) BMI: N/A	Fatigue, headache, insomnia, dysgeusia, hair loss, poor concentration, dizziness, tiredness, fever, and brain fog	Serum Liposome immunometric assay and CH50 hemolytic assay	3 months (90 days) after infection	Patients with LC and post-COVID brain fog had ↑ CH50 levels than those with LC without post-COVID brain fog.
Hurler et al. (2024) [21] United Kingdom	Prospective cohort Healthy controls (n = 47) COVID-19 patients (n = 215) With LC (n = 32)	March 2020–December 2020	Controls 42.3 ± 15.0 y 44.7% females COVID-19 patients 52.9 ± 17.8 y 45.1% females	Hospitalized COVID-19 severity: Moderate (n = 103) Severe (n = 112) BMI: N/A	Fatigue, dyspnea, cough, pain, cognition and memory impairment, neurologic problems, and muscle weakness	Plasma ELISA	3 months (90 days) after infection	No differences in C1-INH, C1s/C1-INH complex, C1q and pentraxin 3 (PTX3) levels among patients with LC, without LC, and controls.
Klein et al. (2023) [19] United States	Case–control Healthy controls (n = 40) Without LC (n = 39) With LC (n = 99)	Not Reported	Controls 36.7 ± 10.2 y 67% females Without LC 38.2 ± 11.7 y 68% females With LC 45.8 ± 13.2 y 68% females	Hospitalized COVID-19 severity: Mild (n = 123) Hospitalized (n = 15) BMI: N/A	Constitutional, neurological, pulmonary, musculoskeletal, gastrointestinal, cardiac, endocrine, ear, nose, throat, and sexual dysfunctions	Blood Immunoassay and plasma proteomics	One year after infection	↑ C4b levels between subjects with and without LC.
Liew et al. (2024) [20] United Kingdom	Case–control Without LC (n = 233) With LC (n = 424) Gastrointestinal LC (n = 132) Fatigue LC (n = 384) Cardiorespiratory LC (n = 398) Cognitive LC (n = 61)	2020–2022	Without LC 58.9 ± 13.7 y 27% females Gastrointestinal LC 57.7 ± 11.5 y 53% females Fatigue LC 56.6 ± 11.1 y 47% females Cardiorespiratory LC 57.1 ± 11.4 y 43% females Cognitive LC 59.2 ± 12.8 y 42% females	Hospitalized COVID-19 severity: No oxygen support (n = 133) Oxygen support (n = 353) Critical care (n = 171) BMI: N/A	Fatigue, anxiety, depression, cardiorespiratory, gastrointestinal, and cognitive problems	Plasma Olink Explore 384 Inflammation Panel	6 months after infection	↑ C1qA levels in gastrointestinal and cognitive LC groups. ↑ COLEC12 (alternative pathway activator) in fatigue and cardiorespiratory LC groups.
Song et al. (2024) [24] China	Prospective cohort Healthy controls (n = 10) COVID-19 patient (n = 50) 1m post-infection (1P1M = 13) 3m post-first infection (1P3M = 9) 6m post-first infection (1P6M = 6) 1m post-second infection (2P1M = 10) 3m post-second infection (2P3M = 7) 6m post-second infection (2P6M = 5)	January 2023– December 2023	Controls 24.6 ± 4.5 y 50% females COVID-19 patients 1P1M 30.9 ± 6.7 y 69.2% females 1P3M 34.9 ± 6.7 y 100% females 1P6M 36.2 ± 7.6 y 66.7% females 2P1M 37.6 ± 13.9 y 90% females 2P3M 39.3 ± 7.7 y 57.1% females 2P6M 37.2 ± 6.5 y 60% females	COVID-19 severity: N/A BMI: N/A	Fever, cough, sore throat, fatigue, anosmia, ageusia, nasal congestion, runny nose, conjunctivitis, myalgia, diarrhea, and dyspnea	Serum/Plasma Nano-LC-MS/MS and MALDI-TOF MS	1, 3, and 6 months after a first and second infection	↓ C3f gradually after the first infection. ↓ C3f gradually further declined after the second infection. In healthy controls, C3f levels were relatively high, almost close to those after recovery from infection.
Wei et al. (2024) [25] China	Case–control Healthy controls (n = 18) Without LC (n = 17) With LC (n = 15)	Not Reported	Controls 43 ± 4.8 y 45% females Without LC 42 ± 13.5 y 43% females With LC 43 ± 13.2 y 47% females	Hospitalized COVID-19 severity: N/A BMI: N/A	Smell/taste dysfunction, fatigue, shortness of breath, and cognitive dysfunction	Plasma Proteomics and metabolomics analyses	Not reported	Patients with LC showed significant dysregulation in complement cascade (C17QA,C1QC,C3,C4A,C5) compared to recovered patients (without LC).

C1s/C1-INH: complement C1s/C1-inhibitor complex; MBL: mannose-binding lectin; TCC: terminal complement complex. ↑ = increased; ↓ = decreased; LC = long COVID.

**Table 3:** Newcastle–Ottawa quality assessment scale evaluating methodological quality/risk of bias (case–control studies).

Study	Selection				Comparability	Exposure			Score
	Representativeness of Cases	Adequate Case Definition	Selection of Controls	Definition of Controls	Comparability Based on the Design or Analysis	Ascertainment of Exposure	Same Method for Cases and Controls	Non- Response Rate	
Aschman et al. (2023) [17]	★	★	★	★	★	★	★	★	8/9
Baillie et al. (2024) [18]	★	★	★	★		★★	★		7/9
Bulla et al. (2023) [23]	★	★	★	★	★	★★	★		8/9
Fernández-de-las-Peñas et al. (2025) [22]	★	★	★	★	★	★★	★		8/9
Klein et al. (2023) [19]	★	★	★	★	★	★	★		7/9
Liew et al. (2024) [20]	★	★	★	★	★	★★	★		8/9
Wei et al. (2024) [25]	★	★	★	★	★	★	★		7/9

**Table 4:** Newcastle–Ottawa quality assessment scale evaluating methodological quality/risk of bias (cohort or longitudinal studies).

Study	Selection				Comparability	Outcome			Score
	Representativeness of Cases	Selection of Non- Exposed Cohort	Ascertainment of Exposure	Outcome of Interest was not Present at Start of Study	Comparability Based on the Design or Analysis	Assessment of Outcome	Follow-Up Long Enough for Outcomes to Occur	Adequacy of Follow-Up	
Hurler et al. (2024) [21]	★	★	★		★★	★		★	7/9
Cervia-Hasler et al. (2024) [12]	★	★	★	★	★	★	★	★	8/9
Song et al. (2024) [24]	★	★	★	★	★	★		★	7/9
Hagiya et al. (2024) [16]	★	★	★	★	★	★	★	★	8/9

**Table 5:** Summary of complement pathway alterations reported in long COVID.

Classical Pathway	Alternative Pathway	Lectin Pathway	Terminal Pathway
Global Post-COVID symptomatology Long COVID *vs.* No post-COVID symptoms (Controls)			
↑ levels C2, C5, C4a, 4b [12,17,19] No differences in C3, C4, C4d, C5 [12,18,22] ↑ levels C1s-C1INH [18] No difference C1s-C1INH [21] C1q: ↑ levels [17] ↓ levels [18] No difference [21]	↑ levels Ba, iC3b, Factor D, Properdin [18] No difference Factor B [18] ↓ levels C3f [24]	No difference MBL [12] No difference MASP1-C1INH complex [18]	↑ levels C5bC6 [12] ↑ levels C5a, C9, TCC [18] ↓ levels C7 [12] No difference C7 [22]
Specific post-COVID symptomatology			
↑ levels C2 in people with post-COVID fatigue [12] ↓ levels C3, C5 in people with post-COVID fatigue [22] ↑ levels C1qA in people with gastrointestinal or cognitive post-COVID symptoms [20] ↑ levels CH50 in people with post-COVID brain fog [16]	↑ levels COLEC12 in people with post-COVID fatigue and cardiorespiratory symptoms [20].	↓ levels MBL in people with post-COVID brain fog [23]	↓ levels C7 in people with post-COVID dyspnea [22]

C1s/C1-INH: complement C1s/C1-inhibitor complex; MBL: mannose-binding lectin; TCC: terminal complement complex. ↑ = increased; ↓ = decreased; LC = long COVID.

## Data Availability

The original contributions presented in this study are included in the article.

## Supplementary Materials

The following supporting information can be downloaded at: https://www.mdpi.com/article/10.3390/biomedicines14020439/s1 [37].
